# Regenerated Corneal Epithelium Expresses More βIII-Tubulin After Chemical Injuries Compared to Mechanical Injuries

**DOI:** 10.1167/tvst.12.12.12

**Published:** 2023-12-12

**Authors:** Athar Shadmani, Ozlem Ercal, Salih Uzun, Aditi Swarup, Albert Y. Wu

**Affiliations:** 1 Department of Ophthalmology, Stanford University School of Medicine, Stanford, CA, USA

**Keywords:** corneal epithelial regeneration, corneal nerve ending, alkali injury, ethanol injury, AlgerBrush injury, corneal wound healing

## Abstract

**Purpose:**

Defining the regenerative response following various types of corneal chemical and mechanical injuries is important for understanding the pathophysiology of the injury and evaluating the effectiveness of the therapies. This study characterizes corneal epithelial healing in a murine chemical and mechanical injury model.

**Methods:**

Four groups of 10 mice each received complete corneolimbal injuries by AlgerBrush, AlgerBrush/thermal, NaOH (0.5 N), or ethanol. Slit-lamp and optical coherence tomography examinations were performed daily for 14 days. Corneal opacity (CO) and neovascularization (NV) were evaluated. The origin of the regenerated epithelium was illustrated by anti-cytokeratin 12 (K12) and anti-K13. The height of regenerated corneal epithelium and intraepithelial free nerve endings (FNEs) stained with anti–βIII-tubulin were measured. The amount of fibrosis was measured by anti–α-smooth muscle actin (α-SMA) monoclonal antibody in the different groups. Statistical analysis was performed by ANOVA and *t*-test.

**Results:**

Corneal opacity and neovascularization were markedly higher in the NaOH and AlgerBrush/thermal groups. Molecular studies revealed the following: Regenerated corneal epithelium thickness was less than normal in all groups, the AlgerBrush group had the shortest height of the regenerated epithelium, βIII-tubulin was expressed in the entire height of corneal epithelium in all groups except in the AlgerBrush group, and K12 was replaced by K13 in all groups.

**Conclusions:**

Corneal wound healing is more effective following chemical injuries in terms of epithelial thickness. Inflammation may play an important role in the outcome.

**Translational Relevance:**

Inflammation following different injuries may be redirected to be more effective in corneal regeneration and clarity.

## Introduction

The cornea is a unique part of the eye that maintains eye shape and accounts for two-thirds of its optical power.^1^ The consistency, transparency, and unique curvature of the cornea are the result of multiple complex interconnected mechanisms. Although the cornea regenerates following minor injuries,^2^ different mechanical and chemical injuries to the cornea and limbus can disrupt these mechanisms and destroy the limbal stem cell niche, which contains stem cells and is densely innervated.^3^ Limbal stem cell deficiency (LSCD) may result in conjunctivalization, corneal opacity (CO), neovascularization (NV), and corneal blindness in severe cases.^4^

Corneal blindness is the second leading cause of preventable eye blindness in the world,^5^^,^^6^ and it places a large socioeconomic burden on society. Critical to addressing this issue are effective prevention strategies and treatments, the development of which can be aided by developing an understanding of corneal biology by utilizing appropriate in vivo injury models.

Alkali injury, which is frequently used in animal studies, results in deep tissue destruction and inflammation.^7^ Basic alkaline compounds are more lipophilic, which allows them to penetrate and damage the eye extensively and induce inflammation in the deep structures of the anterior segment, such as the trabecular meshwork, ciliary body, lens, or even retina.^8^^–^^11^

Ethanol is another popular agent for inducing chemical injury in animal models. It causes cell lysis, proliferation suppression, and apoptosis. Following ethanol exposure, specific corneal epithelial cell marker expression decreases, and corneal epithelial and stromal proinflammatory cytokines and chemokines increase.^12^

Mechanical injury models are commonly created using an AlgerBrush (Alger Company, Lago Vista, TX), which is an effective and easy-to-handle rotating burr tool.^13^ It has been used to debride the corneal and limbal epithelium and create LSCD in multiple studies.^14^^–^^16^ To induce a more stable injury, some studies have combined the AlgerBrush with thermal injury in a mouse animal model.^15^

All injuries initiate corneal inflammation and regeneration; however, inflammation and LSCD severity differ with various injuries. The injury type is a determining factor in the severity of CO, NV, and regenerated epithelial thickness. It is important for clinicians and researchers to be aware of these variations to manage different injuries appropriately and select the injury model targeted to the purpose of their study. To the best of our knowledge, we are the first group to explore and describe clinical and molecular differences between mechanically and chemically induced LSCD models. This study aims to advance our knowledge of corneal regeneration, minimize the number of required animals, and maximize data acquisition. We analyzed clinical and histological LSCD signs, and here we discuss specific features of the different injury models.

## Methods

### Animal Studies and Ethics Statement

Male and female C57BL/6 mice, 8 to 12 weeks old, were kindly provided by the laboratory of Irving L. Weissman (Stanford Medicine). All procedures were carried out in accordance with the Stanford Laboratory Animal Care program (33420), the Care and Use of Animals for Scientific Purposes principles, and the ARVO Statement for the Use of Animals in Ophthalmic and Vision Research. Animal care included a 12-hour cycle of light and darkness and free access to water and food.

### Animal Models and Surgical Methods

**Figure 1.**
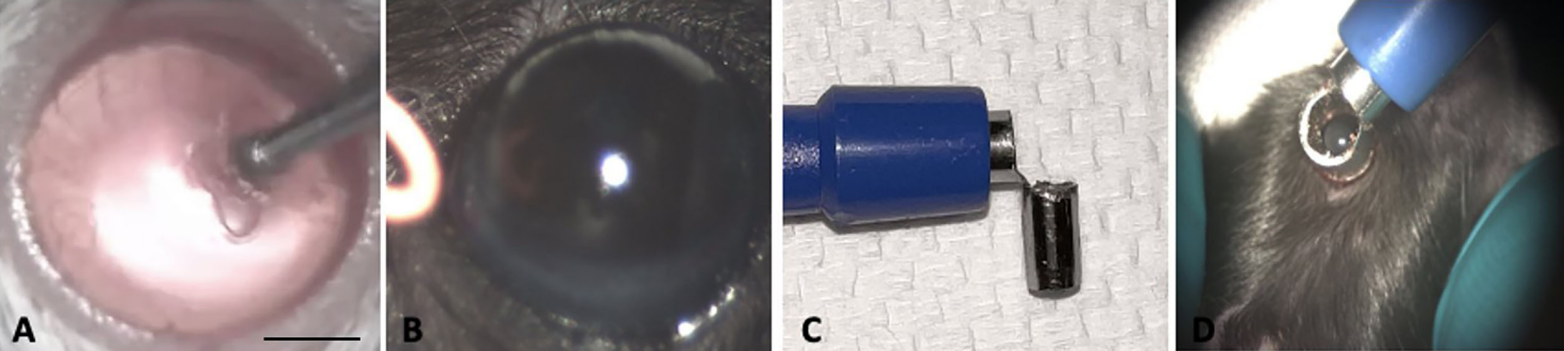
Different mechanical and chemical corneal injuries. (**A**) AlgerBrush is a rotating burr that is used for mechanical debridement of the corneal epithelium and Bruch's membrane (BM). (**B**) To induce more inflammation, thermal injury with surgical cautery in three different limbal areas was added to the AlgerBrush injury. (**C**) A trephine punch based on a 3.5-mm biopsy punch was used to induce an evenly distributed chemical injury to the cornea and limbus. (**D**) The trephine punch was gently held parallel to the axis of the eye, without applying any downward pressure. Three drops of NaOH or ethanol solution were dropped into its hole. The procedure was performed under a surgical microscope. *Scale bar*: 1 mm.

We divided the animals into four groups of 10 mice each; each group received right eye injuries caused by the AlgerBrush, the AlgerBrush with thermal injury, sodium hydroxide (NaOH), or ethanol (Fig. 1). All animals were weighed before anesthesia, which was induced by a mixture of ketamine 100 mg/kg and xylazine 5 mg/kg injected intraperitoneally into the inferior abdominal quadrants.^17^^,^^18^ All eyes were inspected under a slit lamp before the experiments were conducted to verify the absence of any previous ocular injury. The surgery was performed by an ophthalmic surgeon (A.Sh.). In all of the injury models listed below, we applied 2-mm strips of tetracycline and neomycin ophthalmic ointment to the corneal surface only once, immediately following injury induction, to prevent wound infection.^19^

#### AlgerBrush Injury

The entire limbal and corneal epithelium was debrided using a sterile AlgerBrush with a 0.5-mm rotating burr by applying minimal pressure at 45° between the corneal surface and AlgerBrush tip. The rotating burr was moved over the entire corneal and limbal surface, gently and evenly, for 2 minutes (Fig. 1A).

#### AlgerBrush With Thermal Injury

In this group, AlgerBrush injury was followed by thermal injury, which was applied by surgical cautery. First, we induced AlgerBrush injury to the entire corneal and limbal surface for 1 minute, and then we applied thermal injury. After heating the cautery until the tip was red, we retracted the lids away from the limbus with the thumb and index fingers. The cautery was then held at a 5-mm distance from the limbus at the 2, 6, and 10 o'clock positions until shrinkage of the limbus was observed. The tip of the cautery did not touch the limbal area and was kept next to the limbal area for almost 2 seconds (Fig. 1B).

#### NaOH Injury

Because the murine eyeball has a small diameter, the cornea has high curvature to focus light on the retina. Induction of a uniform injury to this highly curved surface by flat filter paper is difficult and unreliable.^20^^,^^21^ To overcome this difficulty, we innovated a simple trephine punch device from a biopsy punch to induce a reproducible and evenly distributed chemical injury as previously described.^22^ The trephine punch was placed down around the mouse limbus, and its hole was filled with 0.5-N NaOH (Figs. 1C, 1D). After 30 seconds, the cornea and fornix were washed with 5 to 8 mL phosphate-buffered saline (PBS) until the pH reached 7 to 7.5.

#### Ethanol Injury

Ethanol injury was induced in the same way as NaOH injury. The cornea was exposed to absolute ethanol for 30 seconds and then washed with 5 mL of PBS.

### Clinical Assessment

CO, epithelial integrity, and NV were clinically evaluated by an ophthalmologist (A.Sh.) using a slit-lamp biomicroscope (SL-450; NIDEK, San Jose, CA) at 0, 2, 4, 6, 8, 10, and 14 days after injury induction. Corneal epithelial integrity was assessed by administering 1 drop of 0.1% fluorescent eye drops prepared from 10% AK-Fluor fluorescent liquid (Long Grove Pharmaceuticals, Rosemont, IL) to the inferior conjunctival sac. Ocular surface photography was performed using an iPhone camera (Apple, Cupertino, CA) in Cinematic mode. Re-epithelialization, fibrosis, and the extent of inflammation were evaluated by histology and immunohistochemistry (IHC) evaluation. CO was scored two times in a masked manner according to the grading system of Yoeruek et al.^23^: 0 = normal, clear; 1 = slightly hazy; 2 = moderately opaque with iris and lens still detectable; 3 = severely opaque with iris and lens barely visible; and 4 = completely opaque with no view of the iris and lens. Corneal NV was graded in accordance with Bahar et al.^24^ with modifications. Limbal NV was restricted to the limbus, paracentral NV extended from the limbus to the periphery of the cornea, and central NV extended to the pupillary area within a 1-mm diameter of the central cornea. SPECTRALIS optical coherence tomography (OCT; Heidelberg Engineering, Heidelberg, Germany) examinations were performed to evaluate the cornea and anterior chamber at 0 and 14 days after surgery.

### Histology

Eyes were enucleated for histopathology on days 0, 1, 4, 7, and 14 after injury; they were then fixed in 10% formalin and embedded in paraffin. The tissue was sectioned at 6 µm and mounted on a glass microscope. Hematoxylin and eosin and periodic acid-Schiff (PAS) staining were performed. Sections were imaged with an EVOS XL Core Imaging System (Thermo Fisher Scientific, Waltham, MA) in 40× brightfield configuration. The infiltrated fibrocytes in the limbal area—from the end of the ciliary body to the beginning of the multicellular layer of the cornea—were counted and compared with other groups.

### IHC Staining

IHC was performed by fixing the eyes in 4% paraformaldehyde and serial sucrose saturation with 10%, 20%, and then 30% sucrose concentrations. The tissue was embedded in Tissue-Tek O.C.T. Compound (4583; Sakura Finetek, Torrance, CA) and was sectioned at a thickness of 12 mm. Corneas were blocked and permeabilized with PBS containing 0.1% Triton X-100 and 5% bovine serum albumin (BSA) for 90 minutes at room temperature. The cells were then incubated overnight at 4°C with primary antibodies diluted in PBS containing 1% BSA and 0.1% Triton X-100. Primary antibodies were rabbit anti-K13 (lot #3202400), diluted 1:100; rabbit anti-K12 (ab185627; Abcam, Cambridge, UK), diluted 1:100; mouse anti–α-smooth muscle actin (α-SMA) monoclonal antibody (A2547; Sigma-Aldrich, St. Louis, MO), diluted 1:400; and rabbit anti–βIII-tubulin antibody (ab18207; Abcam), diluted 1:500. The appropriate secondary antibodies were applied at a 1:500 dilution. Finally, the eyes were flatmounted with Invitrogen ProLong Gold Antifade Mountant with DNA Stain DAPI (P36935; Thermo Fisher Scientific) and examined with confocal microscopy (FV1000; Olympus, Tokyo, Japan). The corneal epithelial height and βIII-tubulin expression at the center of the corneal epithelium were measured using ImageJ (National Institutes of Health, Bethesda, MD) with slides of two eyes from each group. The corneal fibrosis amount was evaluated by measuring subepithelial α-SMA expression thickness by ImageJ.

### Statistical Analysis

Statistical significance comparing multiple groups with parametric data was determined using one-way analysis of variance (ANOVA) with Prism (GraphPad, Boston, MA). A paired *t*-test was applied to compare the CO between days 7 and 14 in each group. All data are presented as the mean ± standard deviation. Tukey's multiple comparison test was used for additional subgroup analysis. *P* < 0.05 was considered statistically significant.

## Results

### Clinical Findings

#### AlgerBrush and AlgerBrush With Thermal Injury

**Figure 2.**
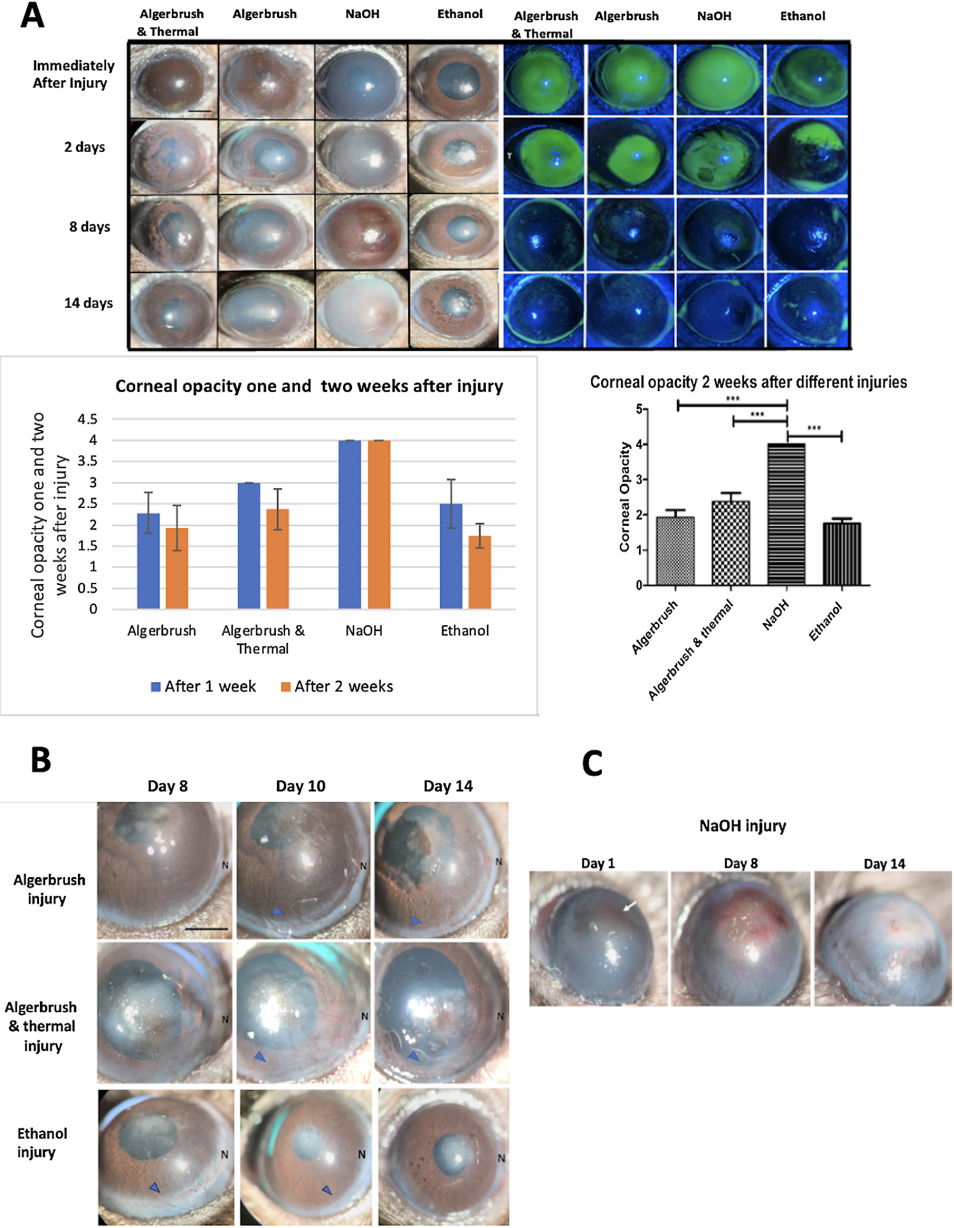
Clinical findings of the cornea and limbus following different injuries. (**A**) Slit-lamp examination revealed a decrease in CO during the second week after injury compared to the first week, except in the NaOH group. The mean CO values after 2 weeks in the AlgerBrush, AlgerBrush/thermal, NaOH, and ethanol groups were 1.9, 2.37, 4, and 1.75, respectively. The re-epithelialization process was slower in the NaOH and AlgerBrush/thermal groups than in the AlgerBrush and ethanol group. Fifty percent of eyes developed persistent epithelial defects by the end of the study. The graphs compare CO 1 and 2 weeks after injury induction. (**B**) During the corneal wound healing, edema gradually decreased, and new vessels formed on the corneal surface, regressed from the healed part of the cornea and localized into the ulcerated areas 2 weeks after the injury (*blue arrowheads*). (**C**) After NaOH injury, intrastromal NVs began to form on the first day after injury induction (*arrow*); they reached their maximum intensity during the first week and gradually decreased, finally being replaced by a thick fibrotic membrane during the second week.

Immediately after these injuries, corneal edema and haze developed. Vibration from the rotating burr, transferred into the small mouse eyes, likely released pigment from the iris and ciliary body into the anterior segment (Figs. 2A, 2B). During the first week, edema continuously decreased from the peripheral to the central part of the cornea and was gradually replaced by fibrosis, which eventually decreased and resulted in a more transparent cornea at the end of the second week. Limbal vessels were dilated and progressively extended their branches to the paracentral and central parts of the corneal surface by days 6 to 8 after injury. During the second week, new vessels were reduced in the healed area and concentrated in the inflamed parts of the cornea (Fig. 2B). In both groups, fibrosis, NV, and pigment deposition were more prominent in the nasal part of the cornea than in other areas (Fig. 2B).

#### NaOH Injury

Immediately after injury, the cornea was edematous with iridocorneal adhesion. Corneal NV started to form in the first 24 hours after injury and was progressively increased until day 8. During the second week, corneal fibrosis extended to the entire cornea and limbus, and the density of NV decreased (Figs. 2A, 2C). Fifty percent of eyes developed persistent epithelial defect at the end of the study.

#### Ethanol Injury

Immediately after 30 seconds of ethanol exposure, the cornea was clear with minimal anterior segment reaction. Corneal edema and haze appeared during the first week; it gradually resolved and was replaced by fibrosis at the center of the cornea by the end of the second week (Fig. 2A).

#### Corneal Opacity and Neovascularization

At the end of the study, the mean CO values in the AlgerBrush, AlgerBrush/thermal, NaOH, and ethanol groups were 1.92, 2.37, 4.00, and 1.75, respectively. CO was clinically decreased compared to the first week in all groups except in the NaOH group. Notably, statistically significant changes in CO levels were observed only in the AlgerBrush/thermal injury group (*P* = 0.039). All NaOH and AlgerBrush/thermal cases and 75% of AlgerBrush cases developed corneal NV. In the AlgerBrush and AlgerBrush/thermal groups, NV was mainly distributed at the nasal part of the cornea, which was more inflamed. The ethanol group showed the lowest NV rate (25%) (Table).

**Table.** Regenerated Corneal Epithelium After Different Mechanical and Chemical Injuries

	Corneal Opacity		NV				
	Mean Score	Distribution	Involved Eyes	Distribution	Fibrocyte Infiltration, *n*	Height of Regenerated Epithelium (µm)	Percent of Regenerated Intraepithelial FNEs to Epithelium
Normal	0	—	—	—	27.7	50	100%
AlgerBrush	1.92	Central and nasal	75%	Nasal and inferior	38.8	28.4	47%
AlgerBrush/thermal injury	2.37	Central and nasal	100%	Nasal	50	36.7	100%
NaOH	4	Diffuse	100%	Diffuse	122	35.3	100%
Ethanol	1.75	Central	25%	Nasal	34.6	34	100%

CO was highest after NaOH injury, which had the highest number of infiltrated fibrocytes. NV was seen in all of the groups; however, the NV rate was least in the ethanol group. The height of regenerated cornea was least in the AlgerBrush group, which had the lowest expression of intraepithelial FNEs.

### OCT Findings

**Figure 3.**
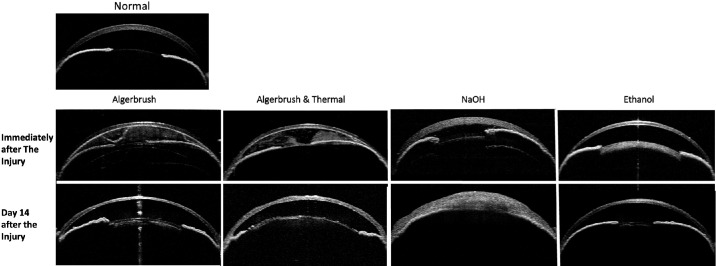
OCT images of normal eyes and eyes after various types of corneal injuries. Immediately after the AlgerBrush and AlgerBrush/thermal injuries, exudate and pigment were released into the anterior segment and were absorbed after 14 days. NaOH induced corneal edema and iridocorneal adhesion immediately after injury, which progressed to severe anterior segment deformity 14 days after injury. Ethanol induced no significant changes in the anterior chamber. N, nasal; T, temporal. *Scale* *bar*: 1 mm. ***P < 0.0001.

OCT examination confirmed pigment and exudate release into the anterior chamber in the AlgerBrush and AlgerBrush/thermal groups that was resolved after 14 days. NaOH injury caused iridocorneal adhesion immediately after injury that progressed to total iridocorneal adhesion and anterior segment deformity. The ethanol group showed the least anterior chamber inflammation and deformation on OCT imaging (Fig. 3).

### Histologic Findings

#### AlgerBrush and AlgerBrush With Thermal Injury

**Figure 4.**
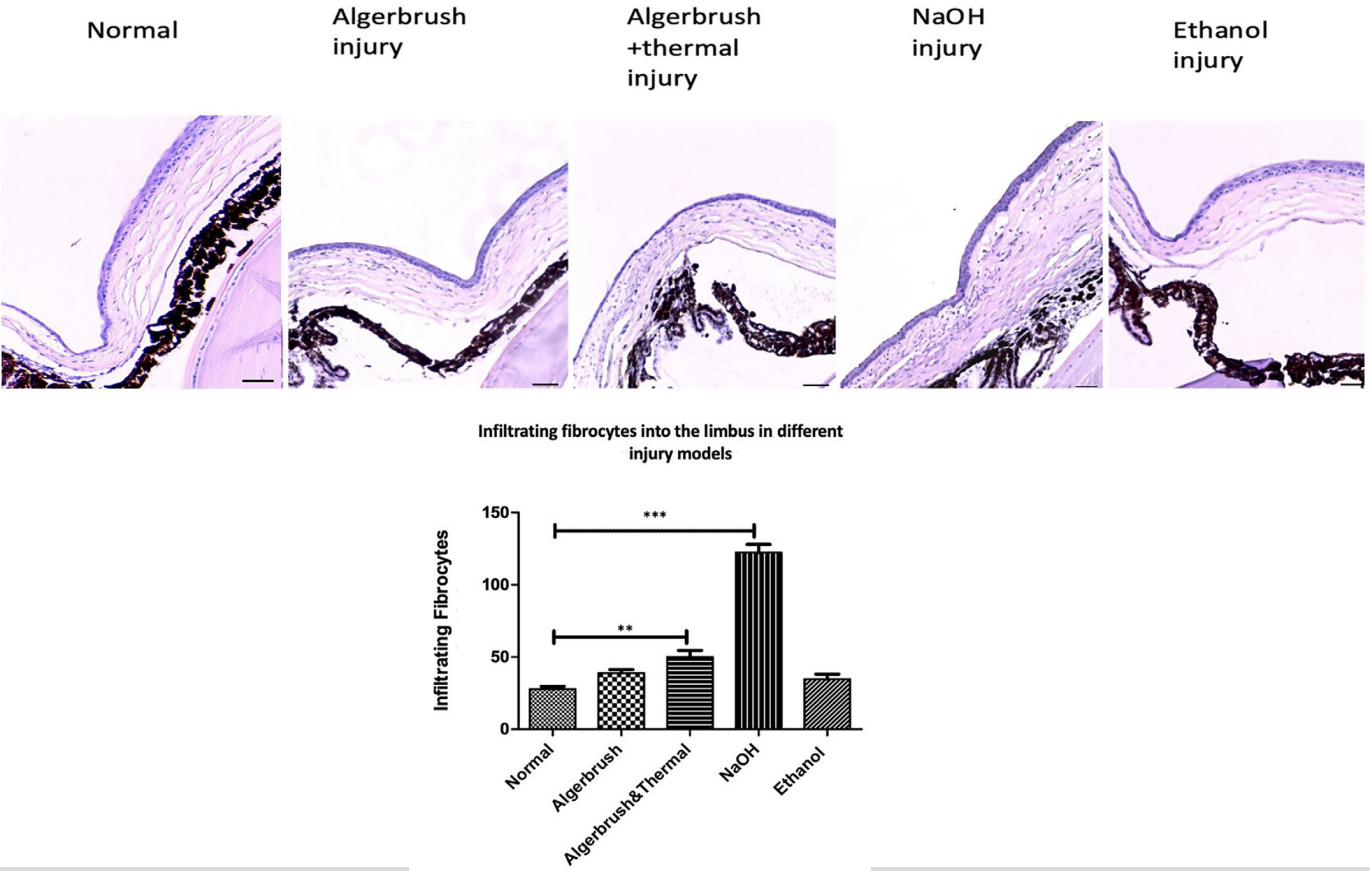
Fibrocyte infiltration into the normal and injured limbus. NaOH-injured eyes had the largest number of cell infiltration, followed by AlgerBrush/thermal injury. The differences among the ethanol, AlgerBrush, and normal groups were not statistically significant. *Scale bar*: 50 µm. ***P ≤ 0.0001. Magnification, 20×.

Immediately after injury, the epithelial cells and Bruch's membrane (BM) were removed with no residual epithelial cells on the corneal surface. On day 4 after injury, the conjunctival epithelium transformed from mono- to multilayers with hyperchromic nucleoli migrating from the peripheral to the central part of the cornea.^25^^,^^26^ The fibrocytes migrated through the limbus to the cornea, where they transformed into keratocytes or myofibroblasts that secreted disordered extracellular matrix (Fig. 4).^27^ In the areas where the epithelium was not regenerated, the BM was poorly recognizable, and epithelial cells were in close interaction with keratocytes.

**Figure 5.**
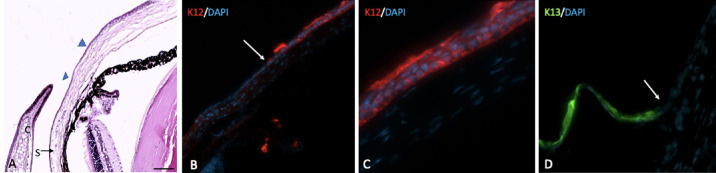
Normal murine cornea and limbus. (**A**) Limbus is the transition from the end of ciliary body to the beginning of cornea, which is covered by an epithelial monolayer (between the *arrowheads*). (**B**, **C**) K12 is expressed by normal corneal epithelial cells from the beginning of cornea. (**D**) K13 is visible only on the conjunctival epithelial cells and cannot be detected on the normal corneal surface (white arrow). S, sclera; C, nasal caruncle. *Scale bar*: 50 µm.

**Figure 6.**
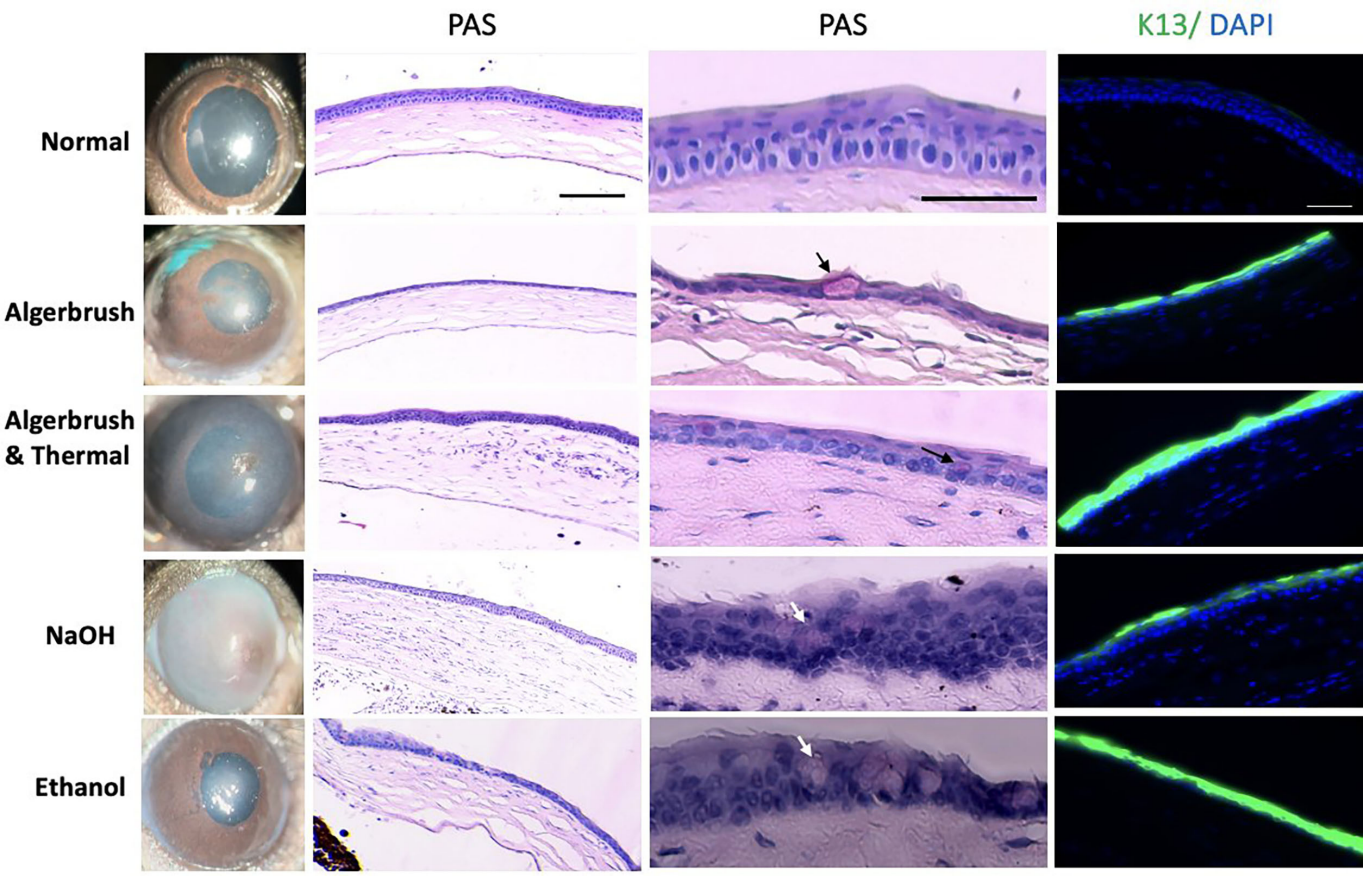
Clinical and histologic findings of the cornea after different injuries. PAS staining illustrated the atrophic regenerated epithelium in the AlgerBrush group. Goblet cells were visible in the PAS-stained peripheral cornea in all injured groups (*arrows*), indicating corneal conjunctivalization. K13, the conjunctival epithelial marker, was not detectable in normal corneas; however, it was expressed with different densities in the regenerated corneal surface. *Scale bar*: 50 µm.

On day 7, two atrophic epithelial layers covered the stroma, which was a reduction compared to the usual four or five layers of normal corneal epithelium (Fig. 5). Goblet cells were present on the corneal surface (Fig. 6). The endothelial cell layer was reactive and exhibited pigment deposition, and the iris was vacuolized with dilated blood vessels.

The number of infiltrated fibroblasts was significantly greater than normal in the AlgerBrush/thermal group (Fig. 4). Goblet cell metaplasia in these groups was less than that in the chemically injured groups. The mean goblet cell numbers in the AlgerBrush and the AlgerBrush/thermal groups were 6.1 and 3.6, respectively.

#### NaOH Injury

Twenty-four hours after injury, the epithelium was absent. The stroma was edematous and hypercellular. The iris was attached to the cornea with dilated vessels and red blood cells extravasating and migrating from the iris vessels into the corneal stroma. Polymorphonuclear cells infiltrated into the stromal tissue from the limbal vessels and iris vessels that adhered to the cornea. Exudate was observed in the anterior chamber. The inflammatory process was more severe in this group than in the AlgerBrush or the AlgerBrush/thermal groups, and the number of infiltrated fibrocytes was greater in this group than in the other groups (Fig. 4). On day 14, the cornea was covered with multicellular layers of epithelial cells that contained the highest density of goblet cells compared to all groups (mean goblet cell number = 14.66 under 40× magnification).

#### Ethanol Injury

The pattern of epithelial cell migration and fibrocyte infiltration to the cornea in this group was like that of the AlgerBrush and the AlgerBrush/thermal groups. Similar to the AlgerBrush-injured eyes, the number of infiltrated fibrocytes was not statistically significant compared to the normal group (Fig. 5). The mean goblet cell number was 9.83, which was the second highest density after the NaOH group.

### IHC Findings

Normal mouse corneal epithelial cells expressed K12, starting from the limbal area through the entire corneal surface. Two weeks after injury, conjunctival epithelial cells covered the corneal surface, which expressed K13, a conjunctival cell-specific marker, throughout all injured corneas. K12 was not expressed in any injured eyes 2 weeks after injury induction (Figs. 5, 6).

**Figure 7.**
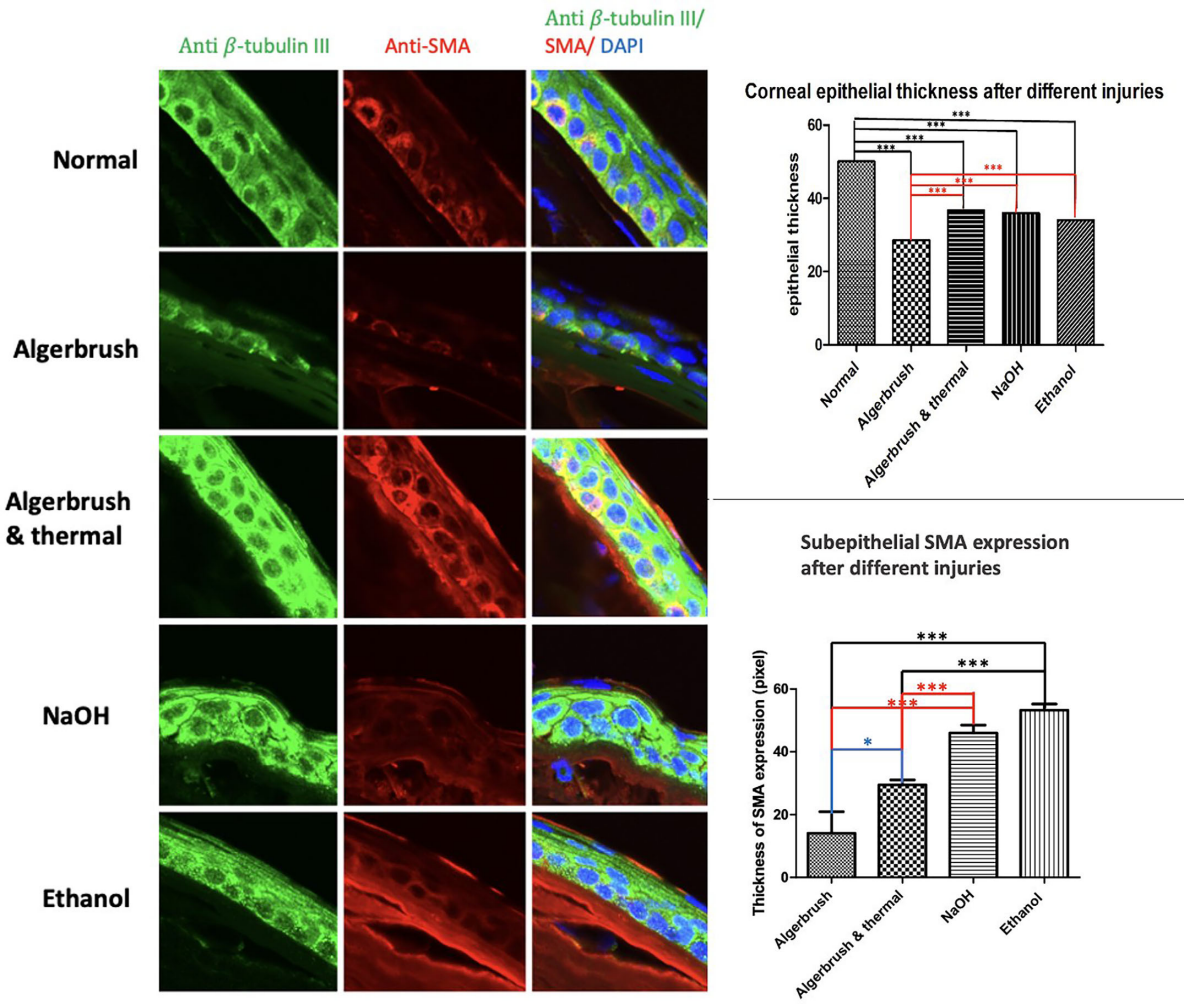
Corneal epithelial regeneration after different injuries. The regenerated corneal epithelial height was less than normal in all injured groups. The AlgerBrush group had the shortest height of regenerated corneal epithelium. βIII-tubulin was expressed in only 47% of the epithelial height but was expressed in the total height of corneal epithelium in all other groups. Subepithelial α-SMA expression, a marker of corneal fibrosis, was highest after NaOH and ethanol injury at the center of the corneal surface. ***P < 0.0001, *P < 0.0497. The images were taken with an Olympus confocal FV1000 microscope.

The regenerated corneal epithelial height was less than normal in all groups (*P* = 0.008). Among all injured groups, the epithelial thickness in the AlgerBrush group was the thinnest compared to the other groups. βIII-tubulin was expressed in the entire thickness of normal and regenerated epithelium in all groups except the AlgerBrush group, where it was expressed in 47% of the regenerated epithelial height (Fig. 7).

Subepithelial fibrosis, estimated by SMA expression at the center of the cornea, was consistent with clinical findings. The highest SMA expression was found in the NaOH and ethanol groups, followed by the AlgerBrush/thermal group. The AlgerBrush-injured eyes expressed the lowest subepithelial SMA marker level (Fig. 7).

## Discussion

Corneal epithelial wound healing is a complex process with a series of interconnected events that are likely orchestrated by corneal nerve endings and inflammatory cytokines. Initial stages of this process involve cell division, inflammation, and re-epithelialization to cover the denuded stroma and restore corneal consistency, and later stages involve restoring corneal clarity via transdifferentiation.^26^^,^^28^

Limbal stem cells proliferate and migrate to cover the denuded stromal surface in injuries limited to the corneal epithelium. However, severe corneal and LSCD result in the destruction of stem cell niches,^29^ resulting in an increase in conjunctival epithelial cell mitotic activity.^25^^,^^30^^–^^32^ This change in the state of cells increases their metabolite and oxygen demand and subsequently causes them to secrete inflammatory cytokines, resulting in the amplification of transforming growth factor β (TGF-β) signaling pathways, which exacerbate inflammation and recruit macrophages and neutrophils to the corneal stroma.^33^ New blood vessels are formed secondary to increasing vascular endothelial growth factor (VEGF) secretion.^34^^–^^38^ This process continues until the denuded corneal surface is covered with conjunctival epithelium.

As the corneal healing process progresses, the rate of mitosis in conjunctival tissue decreases, as does the metabolic demand of the cornea, resulting in a reduction in inflammatory cytokines and the disappearance of newly formed vessels (Figs. 2B, 2C). Epithelium and basement membrane regeneration causes a reduction in TGF-β production and penetration into the stromal tissue. TGF-β is an essential factor for myofibroblast survival,^27^^,^^39^^,^^40^ and a decrease in its concentration results in myofibroblast apoptosis and fibrosis resolution.^41^ Accordingly, the severity of CO decreased during the second week of follow-up in most groups, except in the NaOH-injured group (Fig. 2A).

Corneal epithelial cells are closely related with intraepithelial free nerve endings (FNEs) and the subbasal nerve plexus, which were stained by anti βIII-tubulin.^42^^,^^43^ During corneal wound healing, these nerve fibers, in the form of sensory, sympathetic, and parasympathetic axons, play an important role in controlling epithelial cell regeneration. Sensory nerve endings enhance the adhesion between corneal epithelial cells and improve regenerated epithelial cell maintenance by producing neural-derived growth factors,^6^^,^^44^^–^^47^ whereas the sympathetic fibers increase and the parasympathetic fibers decrease inflammation.^48^

Consistent with these findings, we showed that the AlgerBrush group, which exhibited the shortest epithelial height, expressed the βIII-tubulin marker only in 47% of the regenerated epithelial height. The relationship between intraepithelial FNEs, corneal inflammation, and fibrosis, however, is complex and is unlikely to be direct or linear. Our observations showed that, although the regenerated corneal nerve was prominent after NaOH injury, the CO was the highest in this group. This finding can be explained by the presence of more sympathetic nerve endings in the corneal surface in this injury group. Distinguishing the type of intraepithelial nerve ending was beyond the scope of this study. There was a discrepancy between clinically scored CO and corneal fibrosis, evaluated by subepithelial SMA expression, because in clinical evaluations the entire cornea is used for the evaluation, and the score indicates a mean CO of the entire corneal surface; however, for IHC evaluation, only the center of the cornea is used.

Our study revealed that, similar to human eyes, the nasal part of the mouse cornea is more susceptible to new vessel formation than other parts,^49^ a finding that may be due to fewer subbasal nerves at the nasal cornea compared to the central and temporal regions.^50^^,^^51^ Further studies in this direction with longer follow-up periods are required to gain more insight into this finding.

In conclusion, in this study, we revealed that AlgerBrush injury resulted in less inflammation and intraepithelial FNEs and an atrophic regenerated epithelium compared to chemical injuries, which induced more inflammation, intraepithelial FNEs, and fibrosis. Consistent with other studies, the initial inflammation post-injury may play a significant role in corneal epithelial cell and nerve regeneration by increasing VEGF in the regenerating cornea.^45^^,^^52^ Careful treatment of mechanical and chemical injury cases by reducing inflammatory factors may facilitate the re-establishment of clear corneal epithelium. Further research, with longer observation periods, is necessary to gain a deeper understanding of how providing injured tissue with metabolites and trophic factors may change the inflammation, enhance outcomes, and impact the regenerated corneal epithelium and nerve endings.
